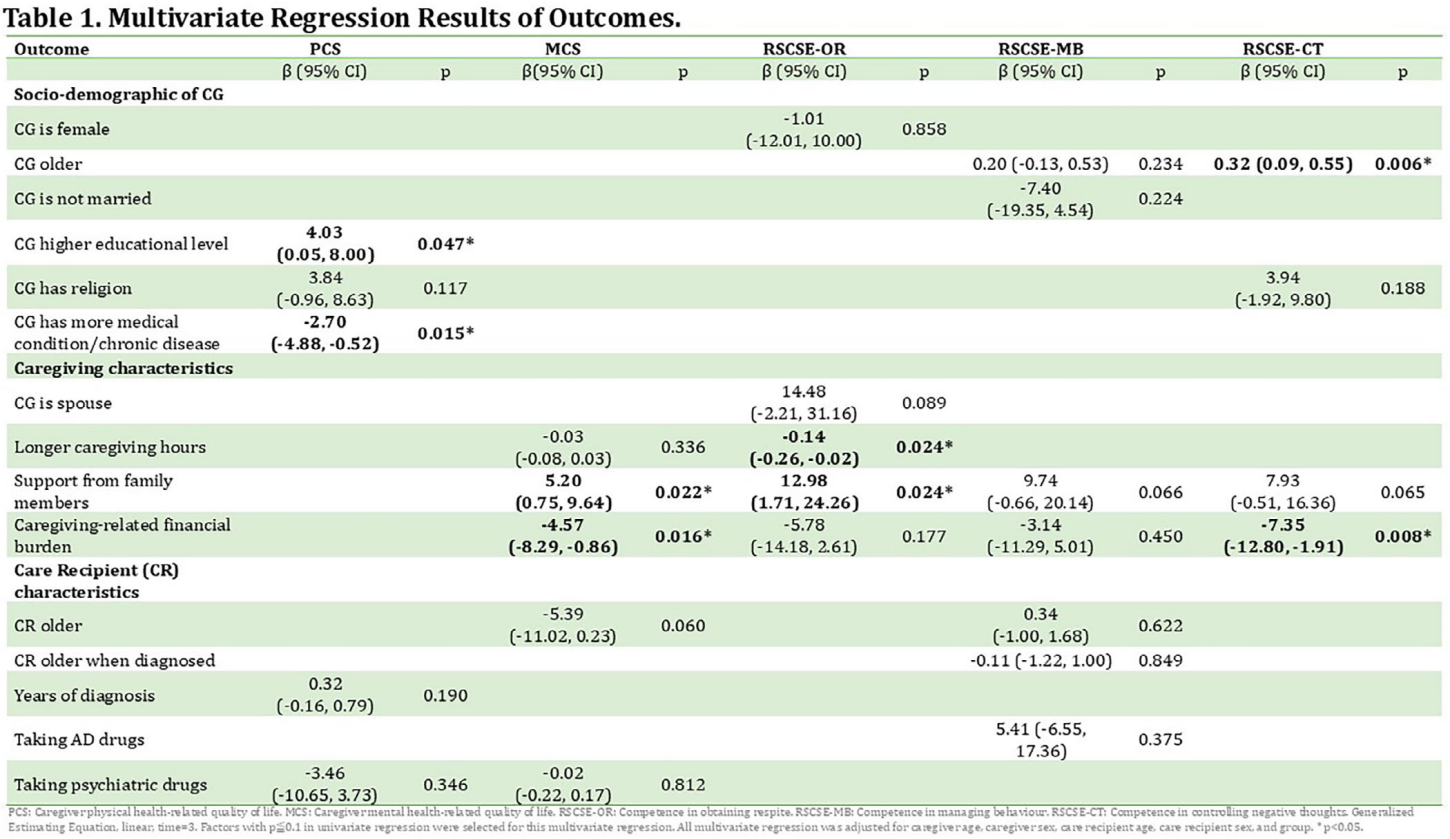# Factors Influencing The Quality Of Life of Carers of People with Dementia: Insights from the iSupport for Dementia Intervention Effectiveness Study

**DOI:** 10.1002/alz70858_101834

**Published:** 2025-12-25

**Authors:** Bel Wong, Lily Dongxia Xiao, Mingxia Zhu, Tobi Cheng, Florence Ho, Timothy CY Kwok

**Affiliations:** ^1^ Jockey Club Centre for Positve Ageing, HK, HK, Hong Kong; ^2^ Flinders University, Adelaide, SA, Australia; ^3^ Kiang Wu Nursing College of Macau, Macau, China; ^4^ Department of Medicine and Therapeutics, The Chinese University of Hong Kong, Hong Kong, China

## Abstract

**Background:**

The carers of people with dementia (PWD) often face physical, emotional, and financial challenges; their well‐being is influenced by various carer‐related and care recipient‐related factors within the stress and process framework. This study aimed to examine the factors associated with the quality of life and caregiving well‐being of dementia carers to better inform the design of supportive interventions and enhance carer support.

**Method:**

This study adopted a longitudinal cohort design. The outcome measures were carers’ physical‐ and mental‐related quality of life, and caregiving self‐efficacy. The data were obtained from the effectiveness study of iSupport for Dementia, an online psychoeducation programme developed by The World Health Organization, conducted in Hong Kong and Macau, and were analysed using Generalized Estimating Equation. This study was funded by the National Foundation for Australia‐China Relations, Australian Government.

**Result:**

Data from 63 carers of PWD were analysed. The results showed that, after adjusting for covariates, carers’ health‐related quality of life was positively associated with carers’ education level (Adjusted odds ratio [AOR]=4.03, 95% CI=0.05 – 8.00, *p* = 0.047) and support from other family members (AOR=5.20, 95% CI=0.75 – 9.64, *p* = 0.022), but negatively associated with carers’ own medical condition (AOR=‐2.70, 95% CI=‐4.88 – ‐0.52, *p* = 0.015) and caregiving‐related financial burden (AOR=‐4.57, 95% CI=‐8.29 – ‐0.86, *p* = 0.016). Higher caregiving time (AOR=‐0.14, 95% CI=‐0.26 – ‐0.02, *p* = 0.024) and caregiving‐related financial burden (AOR=‐7.35, 95% CI=‐12.80 – ‐1.91, *p* = 0.008) were linked to worse caregiving self‐efficacy, while older carer age (AOR=0.32, 95% CI=0.09 – 0.55, *p* = 0.006) and family support (AOR=12.98, 95% CI=1.71 – 24.26, *p* = 0.024) were associated with better caregiving self‐efficacy.

**Conclusion:**

Family support plays a crucial role in enhancing health‐related quality of life and caregiving self‐efficacy, while financial burden negatively impacts carers’ well‐being. iSupport for Dementia emphasises sharing caregiving responsibilities and exploring available support through self‐reflection exercises and practical scenarios. It also includes region‐specific resources during adaptation to help carers access affordable dementia care. Future psychoeducation interventions for carers should consider these factors to address their challenges effectively and provide comprehensive support to enhance their overall well‐being.